# Endoscopic radial incision method for two strictures of the esophagus after endoscopic submucosal dissection: a case report

**DOI:** 10.1186/s12957-020-01812-z

**Published:** 2020-02-13

**Authors:** Zhong Huang, Wei Wei, Fang Cheng

**Affiliations:** Department of Gastroenterology, Zigong First People’s Hospital, 42 Shangyihao Road, Zigong, 643000 Sichuan Province China

**Keywords:** Early esophageal cancer, Endoscopic radial incision, Esophageal stricture, Endoscopic submucosal dissection, Case report

## Abstract

**Background:**

The development of severe esophageal stricture after endoscopic submucosal dissection (ESD) for early esophageal carcinoma is not uncommon. Dilation by Savary-Gilliard dilators or balloon dilators is the first-line treatment for such complex refractory benign stricture, but it has a high risk of treatment failure. So far, endoscopic radial incision (ERI) as a new technology for the treatment of post-ESD esophageal stricture has been rarely reported. We report a case, which we designed to assess the efficacy and safety of ERI technology for two severe strictures of the esophagus after ESD.

**Case presentation:**

A 67-year-old man had suffered from two complex refractory benign strictures of the esophagus after ESD for early esophageal carcinoma. The patient was refractory to multiple endoscopic balloon dilation (EBD) therapy previously. Thus, the patient underwent ERI successfully and without postoperative complications such as fever, poststernal pain, bleeding, and perforation. During 3 months of follow-up after ERI, the patient had no recurrence of dysphagia.

**Conclusions:**

Refractory strictures of the esophagus after ESD are common. ERI is a safe and efficient strategy for treating such multiple refractory esophageal strictures.

## Background

Esophageal cancer is one of the most unknown and fatal malignancies worldwide. Patients with esophageal cancer have low quality of life. So, early detection and treatment is very important. With the development of endoscopic technology, the diagnostic rate of early esophageal cancer is increasing. Endoscopic therapy for early esophageal neoplasms also can result in a minimum number of complications and preserve the esophagus. So, early diagnosis and treatment of esophageal cancer can improve the prognosis of patients. Endoscopic submucosal dissection (ESD) was introduced in Japan to treat gastric neoplasia. With the development of ESD, it is accepted as the main therapeutic approach for superficial esophageal cancer [1, 2]. ESD can be performed for en bloc resection of the lesions and precise histopathological evaluation, and it also can result in low recurrence rates. But, benign stricture after ESD is not an unusual event. The incidence of post-ESD esophageal stricture ranges from 70 to 90%. Patients suffer from dysphagia, nausea, and vomiting, which will decrease the quality of life and increase their economic burden. The exact pathophysiology of the stricture is unknown. Some articles think under the influence of physical and chemical factors, activation of fibroblast proliferation promotes collagen deposition and scar formation, which will lead to contracture of esophagus [3]. Now, the endoscopic treatment of esophageal stricture includes conventional treatment strategies (dilatation, stent insertion, locoregional injection). Among these, the endoscopic balloon dilation (EBD) is the most common and simplest therapy. But, most patients need to undergo multiple dilatations (2-9 times) [4, 5]. The common complications of EBD include bleeding and perforation. Complex strictures are at higher risk of treatment failure and are considered as refractory after repetitive unsuccessful dilations. Particularly, a patient suffers mucosal defects of over 3/4 of the circumference of the esophagus. Therefore, the management of complex refractory benign strictures after ESD has remained a challenging problem. The ERI technique is recently developed to treat such complex refractory benign strictures, and there are several reports that show ERI is effective for esophageal strictures [6, 7]. So, the ERI procedure is a new technique that has been described for the treatment of refractory esophageal strictures. We should evaluate the effectiveness and safety of ERI for benign strictures of the esophagus after ESD. In this paper, we report a case of ERI for the treatment of multiple refractory strictures of the esophagus after ESD successfully.

## Case presentation

**Fig. 1:**
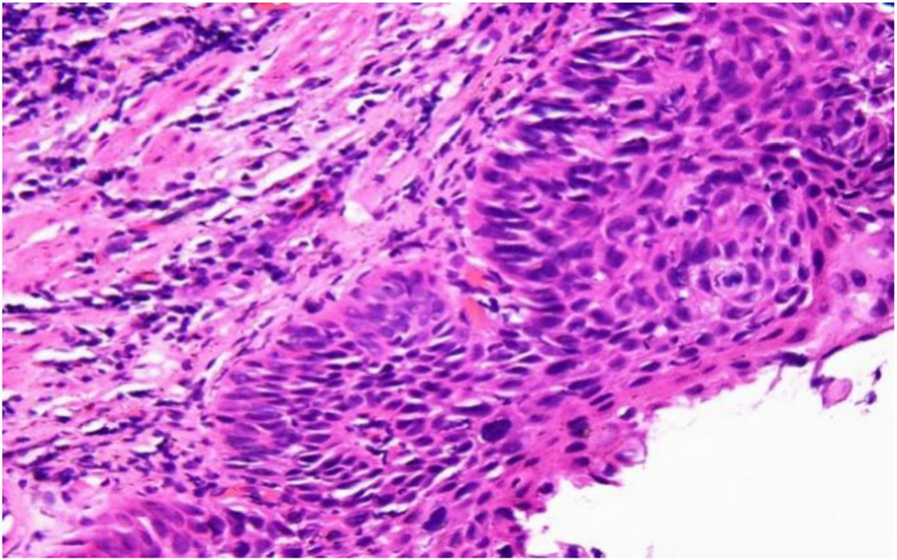
The pathology of the esophagus indicated moderate to severe squamous epithelial dysplasia

**Fig. 2:**
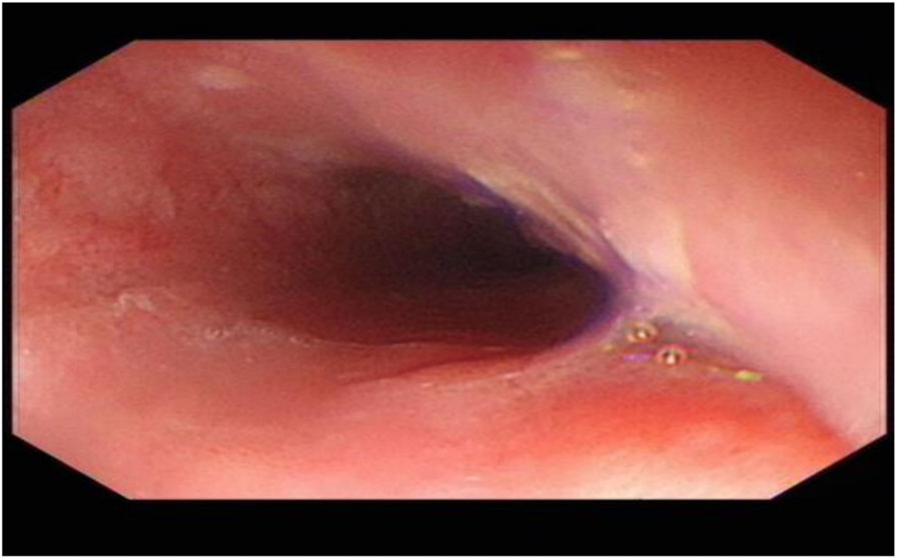
EGD showing early esophageal cancer, 28-33 cm from the incisors

**Fig. 3:**
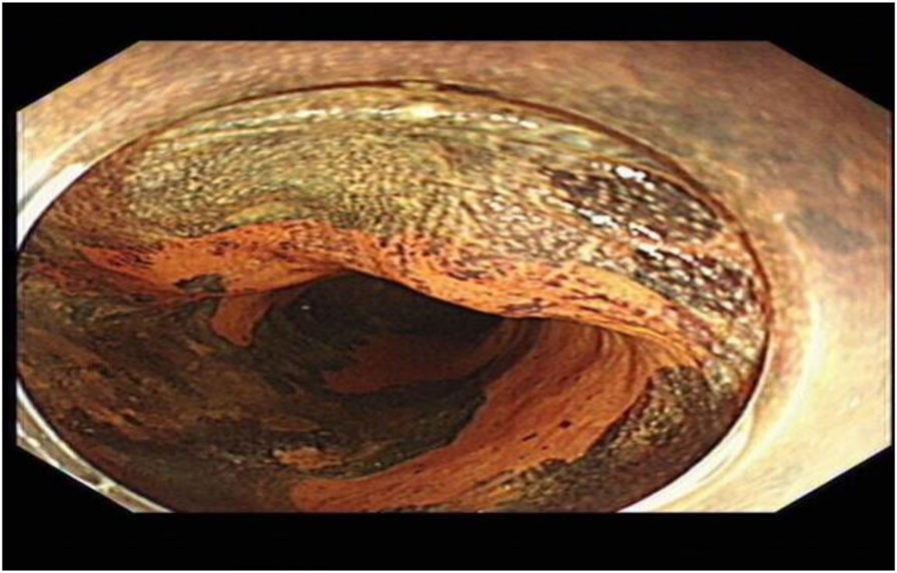
NBI staining for the lesion was brown

**Fig. 4:**
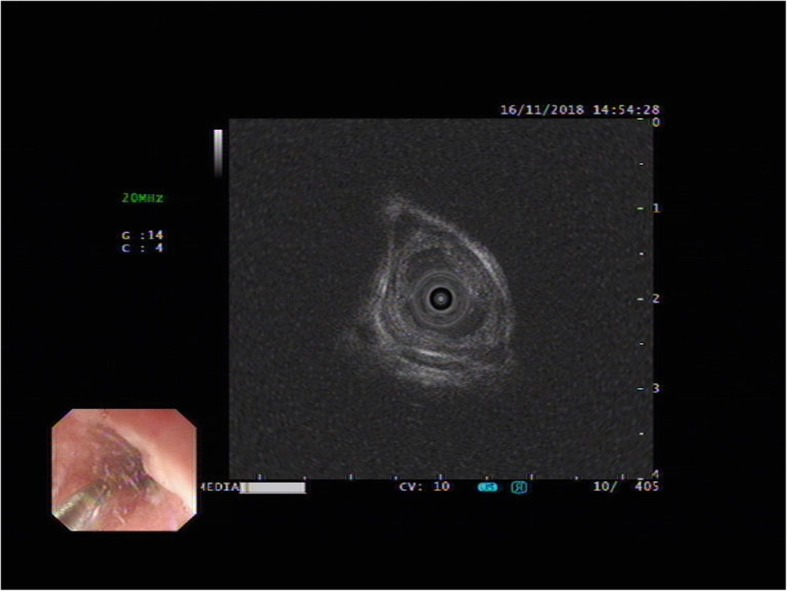
EUS showing for lesion before ESD

**Fig. 5:**
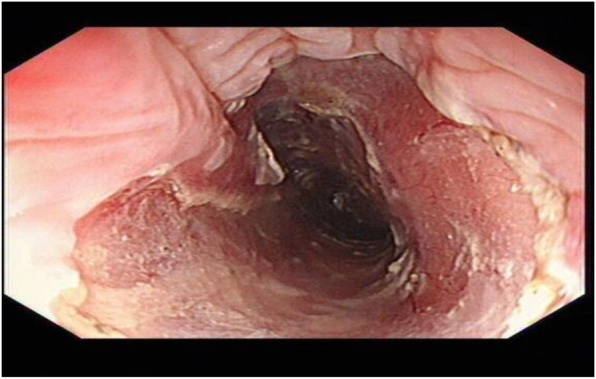
En bloc resection of lesions by ESD

**Fig. 6:**
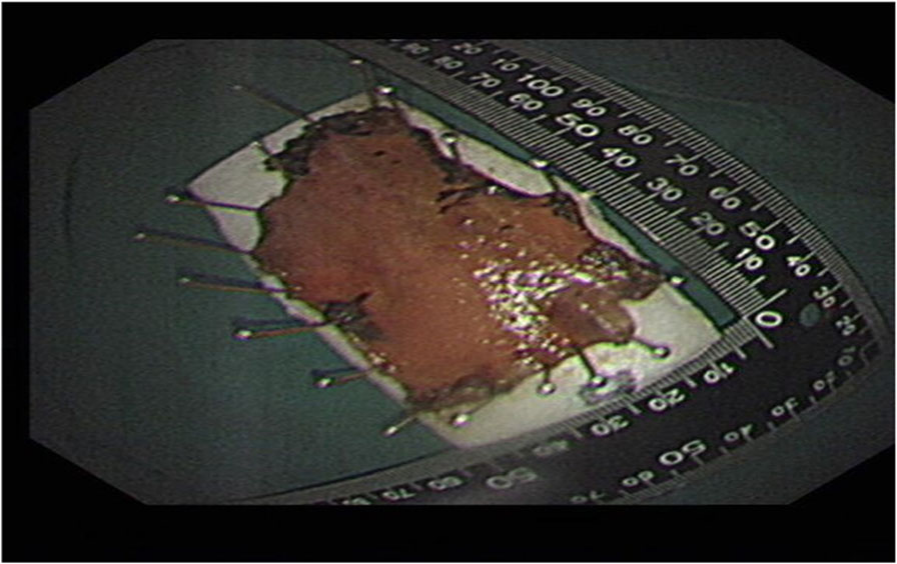
The resected lesion size was 5.0 × 8.0 cm

**Fig. 7:**
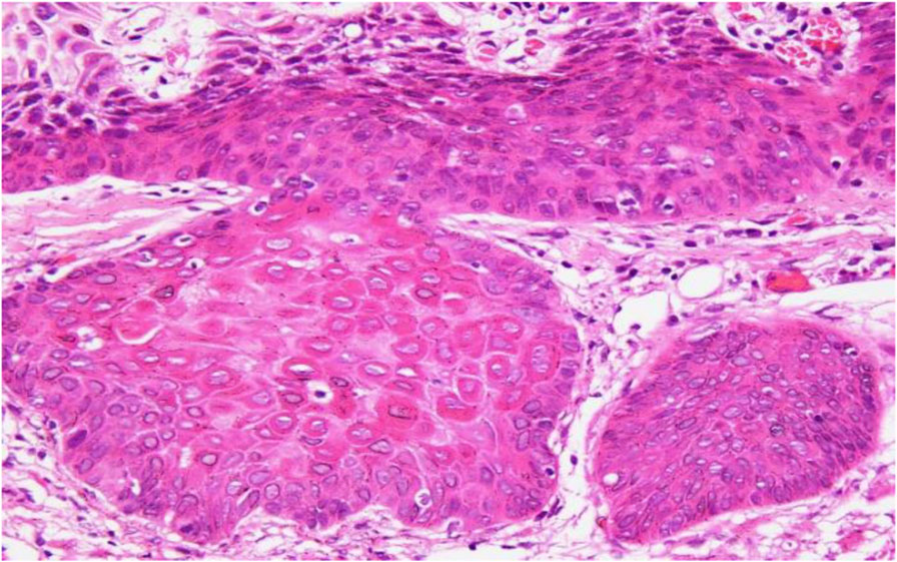
High-grade intraepithelial neoplasia of squamous epithelium; cancer cells infiltrated muscularis propria of the esophagus

A 67-year-old man presented to our department complained of substernal pain for 1 year. EGD showed the esophageal mucosal congestion, erosion, and roughness, 29~31 cm from the incisors. Pathological analysis indicated squamous-cell middle-severe dysplasia of an esophageal mucosa (Fig. 1). We achieved en bloc resection by ESD without adverse events. During the operation, it was shown that the lesion flaky mucosa was rough and the surface structure disorder infiltrated 3/4 circumference of the esophageal lumen, 28~33 cm from the incisors (Fig. 2). Narrow-band imaging (NBI) staining for the lesion was brown (Fig. 3). Ultrasonic gastroscopy indicated that the mucosal lesion was thickened, and there was a clear boundary between the submucosa and muscularis propria (Fig. 4). Therefore, ESD was performed with a HybridKnife, and the primary outcome was en bloc resection of the lesions. There was no bleeding or perforation after wound management with electrocoagulation (Fig. 5). So, ESD for the treatment of early esophageal cancer was feasible and safe. The resected lesion size was 5.0 × 8.0 cm (Fig. 6). The post-ESD pathological analysis was high-grade esophageal intraepithelial neoplastic; cancer cells infiltrated the lamina propria mucosae of the esophagus (Fig. 7). The patient underwent resected lesion with a negative margin. To prevent the formation of the esophageal stricture after ESD, the patient continued to take oral prednisone and intraluminal stent insertion for 30 days. However, the patient developed progressive dysphagia on the seventh day after the removal of the esophageal stent. EGD showed an esophageal stricture. The patient undergone repeated EBD in other hospitals, but it was ineffective. Eventually, the patient was admitted to our department for endoscopic evaluation and therapy, considering the patient had failed multiple EBD. Finally, we chose ERI as the treatment for post-ESD esophageal stricture.

**Fig. 8:**
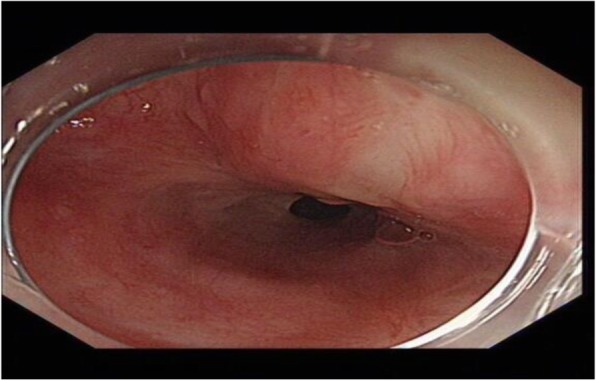
EGD showed a cicatricial stricture of the esophagus (diameter 0.6 cm)

**Fig. 9:**
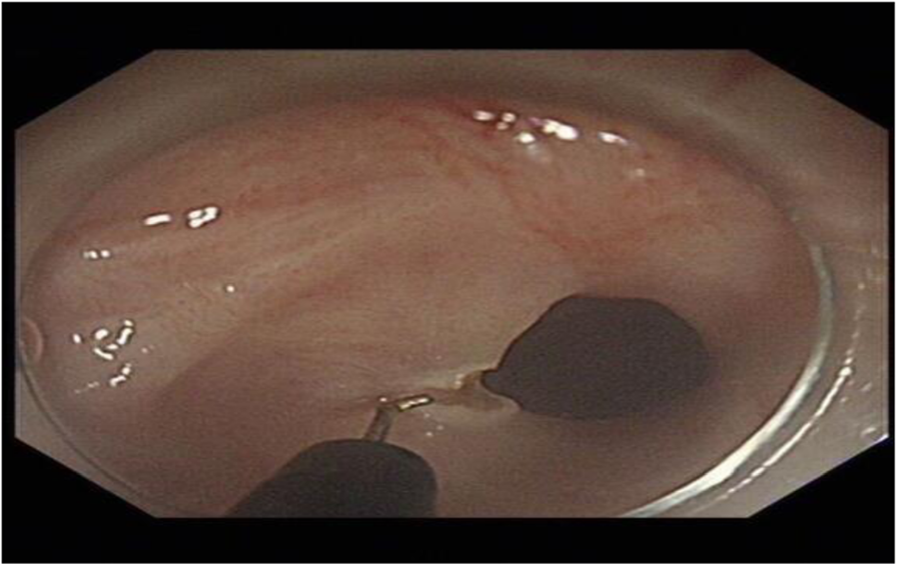
Used the insulated-tip knife to performed ERI

**Fig. 10:**
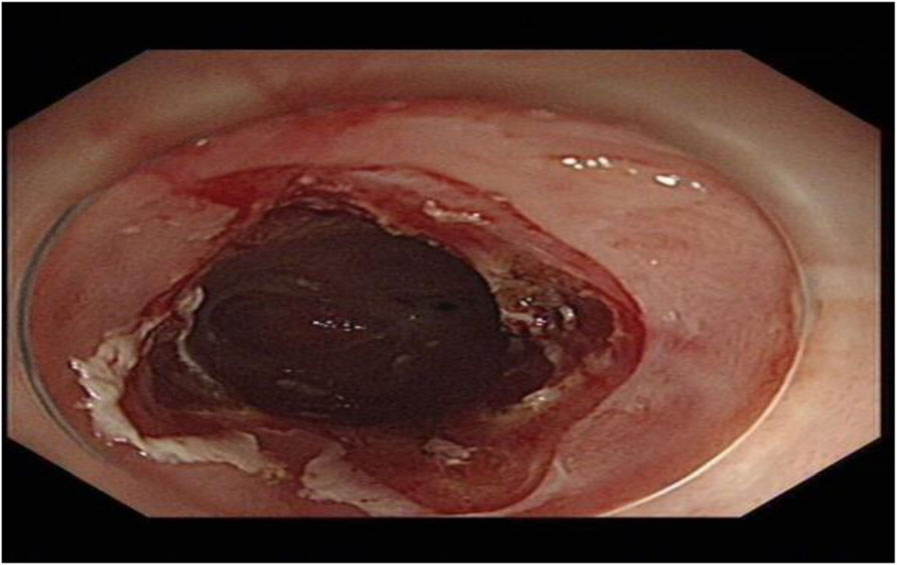
The first stricture. The stricture was removed by ERI (diameter 1.3 cm)

**Fig. 11:**
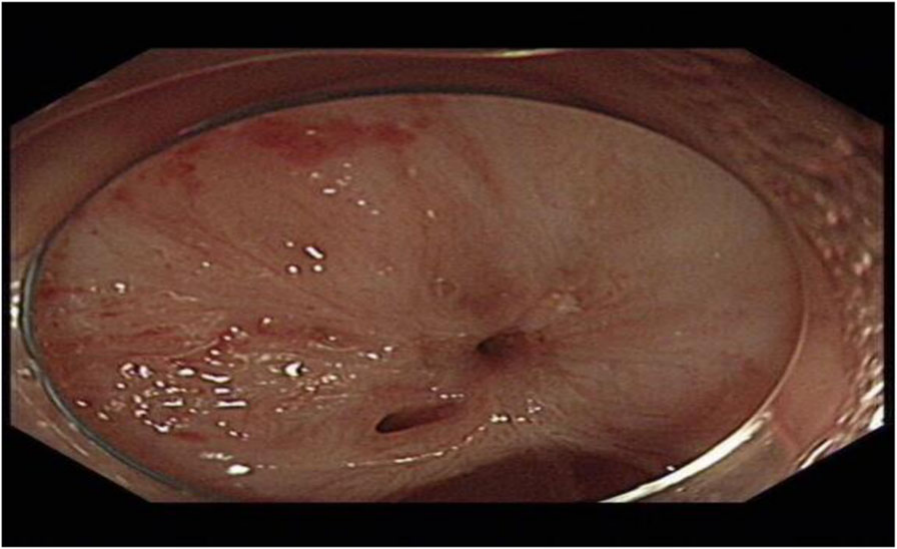
EGD showed another cicatricial stricture of the esophagus (diameter 0.2 cm)

**Fig. 12:**
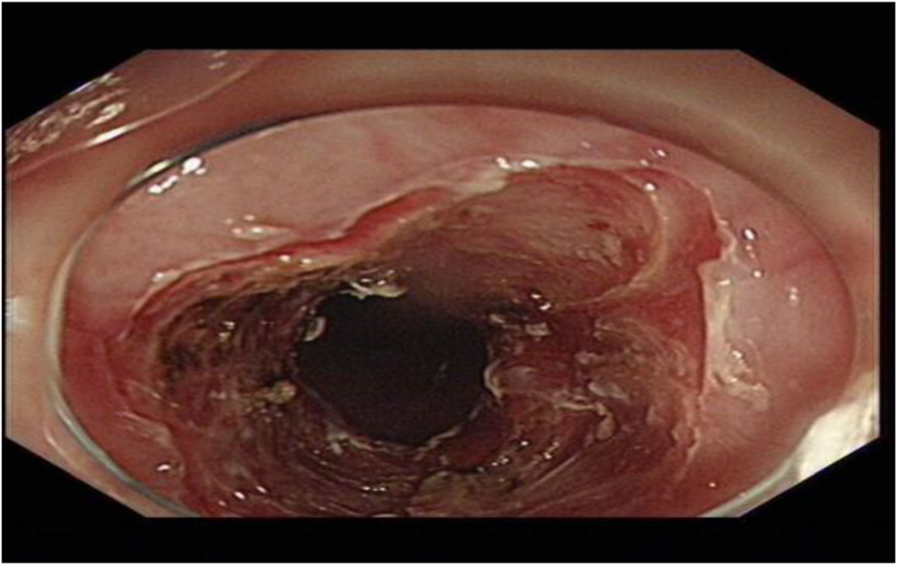
The other stricture. The stricture was removed by ERI (diameter 1.2 cm)

**Fig. 13:**
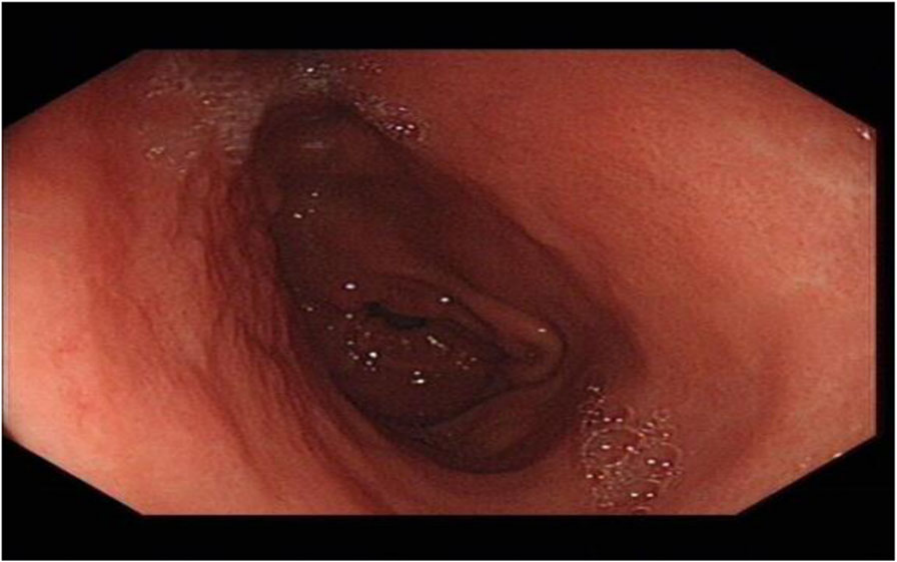
The gastroscope into the stomach without resistance

The patient had an unremarkable physical and systemic examination. No abnormal physical finding or laboratory data were observed. Chest computed tomography (CT) showed thickening of the middle part of the esophagus wall. EGD showed a benign cicatricial stricture (diameter 0.6 cm) of the esophagus, 29 cm from the incisors (Fig. 8). And the gastroscopy cannot pass through smoothly. The NBI showed the surface structure of the esophageal mucosa was regular. We used the insulated-tip knife to perform ERI, and the incision was made to cut the superficial muscle layer along the line that connects the esophageal lumen on the oral side and the lumen on the anal side in the 3, 6, 9, and 12 o’clock direction. The cutting depth ranges from 4 to 6 mm. When the endoscope can pass through the structure, then the stricture of the esophagus was a widespread cutting (Fig. 9). Finally, the symptoms improved (diameter 1.3 cm) (Fig. 10). We used electrocoagulation for hemostasis and without bleeding or perforation. Next, we saw another severe cicatricial stricture (diameter 0.2 cm) of the esophagus, 31 cm from the incisors (Fig. 11). ERI also was performed. Finally, the symptoms improved (diameter 1.2 cm) (Fig. 12). The gastroscope into the stomach without resistance is shown in Fig. 13. The wound was treated with electrocoagulation without bleeding. The procedure took approximately 30 min. The patient with dysphagia was significantly relieved and without complications such as fever, poststernal pain, bleeding, and perforation. We followed up the patient for 3 months and there was no recurrence of dysphagia. Long-term follow-up is ongoing.

## Discussion

ESD has been widely applied as a treatment way for early esophageal cancer because it is minimally invasive and more effective in the en bloc resection. The complications of ESD include bleeding, perforation, and strictures. Some studies showed there is a high rate of post-ESD stricture of the esophagus, and that makes it difficult for patients to swallow which leads to a low quality of life. The stricture rates of the esophagus is up to 70–90% if patients suffer circumferential mucosal defects of more than 3/4 of the circumference of the esophagus [8]. Esophageal scar strictures after ESD often belongs to complex refractory benign strictures [9]. Now, there is no standard treatment for the esophageal stricture after ESD. In recent years, the common treatment way for such complex refractory benign stricture is repeated dilation by Savary-Gilliard dilators or balloon dilators. There was a study that reported that in using oral steroids to prevent post-ESD esophageal stricture, 45% of patients still suffered from stricture [10]. EBD is effective for benign stricture, and it needs to be performed frequently until the dysphagia disappears, although EBD can open the esophageal lumen that cannot have long-term maintenance because of repeated traumatic dilations can increase the formation of mucosal scars [11]. So, some patients are refractory to dilation therapy. We think it is important to prevent the formation of esophageal stricture after ESD, and it is also urgent to find a better method for esophageal stricture. The risk factors and mechanisms of post-ESD stricture of the esophagus have not been fully elucidated. Some studies showed that immune factors, the ratio of the mucosal defect of circumference, the longitudinal length changes in esophageal mucosal fibroblasts, the intraoperative thermal injury, and the scar formation caused by the operation have a great impact on the stricture [12]. Lesions involved lamina propria of the esophageal mucosa, a mucosal defect that exceeds 3/4 of the esophageal circumference, and are the risk factors for esophageal strictures after ESD [13, 14]. Based on these, we think the lowering of elasticity and movement of the esophageal wall is one of the important reasons for post-ESD esophageal stricture. In our case, the patient included all of the above risk factors. Although the patient received oral glucocorticoids and stent insertion post-ESD to prevent esophageal stricture, there were still two severe strictures of the esophagus which were ineffective to balloon dilation. The gastroscope cannot pass through the esophagus, and the patient suffered from severe dysphagia. So, in our present report, we describe the strictures were refractory to conventional EBD. Now, some studies showed ERI is a novel option for refractory rectal/esophageal stricture [6, 7, 15]. Lee et al. [16] evaluated the long-term efficacy of incisional therapy for benign stricture of esophageal anastomotic at the first time; operation-related adverse event has not been reported. Follow-up to 2 years suggested that the effective rate of incisional therapy was 87.5%. ERI was efficient for refractory stricture by slicing off the fibrotic tissues caused by EBD. These evidences show that incisional therapy appears to offer a safe and effective treatment approach for esophageal stricture. In our case, the patient suffered from refractory benign strictures that had been unresponsive to conventional therapy (EBD). We used ERI to relieve esophageal strictures. There is no ERI-related adverse event, and the patient recovered quickly after ERI. Follow-up was done for 3 months without recurrence.

Although there were few reports about the application of ERI for post-ESD esophageal stricture, it is a promising mean of managing recurrent stricture. There are some experiences that are summarized as follows: (1) CT and esophagography before ERI can help us preliminarily know the esophageal strictures and provide a reference for the selection of the incision site during operation. (2) Narrowing of the esophageal lumen due to hard scar tissue formation, it is difficult to inject medium to make a submucosal fluid pad before ERI. Therefore, the risk of perforation is high. We recommend experienced doctors to manage ERI. It is important to confirm the fibrosis to be excised with EUS before ERI and during ERI. However, this time the esophageal stenosis was as small as 0.2 cm, and the EUS probe would not have passed. (3) Cutting from shallow to deep and along the line that connects the esophageal lumen on the oral side and the lumen on the anal side, to ensure the full cut of stricture, we should choose 4~6 directions to cut; (4) operative wound should be treated carefully, such as electrocoagulation; (5) observe postoperative complications closely, such as bleeding, subcutaneous emphysema, fever, and post-sternal pain.

## Conclusions

In conclusion, ERI is a safe and effective therapy for the treatment of esophageal benign stricture and improving patients’ quality of life, especially for complex refractory esophageal stricture after ESD. In the future, with the development of endoscopic technology, more studies are needed to support our conclusions.

## Data Availability

All data generated or analyzed are included in this published article.

## Abbreviations

CT: Computed tomography
EBD: Endoscopic balloon dilation
EGD: Esophagogastroduodenoscopy
ERI: Endoscopic radial incision
ESD: Endoscopic submucosal dissection
EUS: Endoscopic ultrasonography
NBI: Narrow band imaging

## References

[1] Fujishiro M, Yahagi N, Kakushima N, Kodashima S, Muraki Y, Ono S (2006). Endoscopic submucosal dissection of esophageal squamous cell neoplasms. Clin Gastroenterol Hepatol.

[2] Oyama T, Tomori A, Hotta K, Morita S, Kominato K, Tanaka M (2005). Endoscopic submucosal dissection of early esophageal cancer. Clin Gastroenterol Hepatol..

[3] Qi L, He W, Yang J, Gao Y, Chen J (2018). Endoscopic balloon dilation and submucosal injection of triamcinolone acetonide in the treatment of esophageal stricture: a single-center retrospective study. Exp Ther Med..

[4] Lew AJ, Kochman ML (2002). A review of endoscopic methods of esophageal dilation. J Clin Gastroenterol.

[5] Chiu YC, Hsu CC, Chiu KW, Chuah SK, Changchien CS, Wu KL (2004). Factors influencing clinical applications of endoscopic balloon dilation for benign esophageal strictures. Endoscopy..

[6] Mizusawa T, Kobayashi M, Terai S (2019). Radial incision and cutting for refractory benign esophageal stricture. Dig Endosc..

[7] Jia Y, Chen C, Jin P, Yu D, Wang L, Li S, et al. Endoscopic radial incision combined with dexamethasone therapy for stricture of the esophagus caused by Crohn’s disease. Endoscopy. 2019;23. 10.1055/a-0915-1539 31121619

[8] Lewis JJ, Rubenstein JH, Singal AG, Elmunzer BJ, Kwon RS, Piraka CR (2011). Factors associated with esophageal stricture formation after endoscopic mucosal resection for neoplastic Barrett’s esophagus. Gastrointest Endosc.

[9] On OS, Fujishiro M, Niimi K, Goto O, Kodashima S, Yamamichi N (2009). Predictors of postoperative stricture after esophageal endoscopic submucosal dissection for superficial squamous cell neoplasms. Endoscopy.

[10] Tang B, Bai JY, Zhao XY, Fan CQ, Yang X, Deng L (2015). Endoscopic submucosal dissection for superficial esophageal cancer with near-circumferential lesions: our experience with 40 patients. Surg Endosc..

[11] Cheng YS, Li MH, Yang RJ, Zhang HZ, Ding ZX, Zhuang QX (2003). Restenosis following balloon dilation of benign esophageal stenosis. World J Gastroenterol.

[12] Mizuta H, Nishimori I, Kuratani Y, Higashidani Y, Kohsaki T, Onishi S (2009). Predictive factors for esophageal stenosis after endoscopic submucosal dissection for superficial esophageal cancer. Dis Esophagus..

[13] Shi Q, Ju H, Yao LQ, Zhou PH, Xu MD, Chen T (2014). Risk factors for postoperative stricture after endoscopic submucosal dissection for superficial esophageal carcinoma. Endoscopy.

[14] Isomoto H, Yamaguchi N, Minami H, Nakao K (2013). Management of complications associated with endoscopic submucosal dissection/endoscopic mucosal resection for esophageal cancer. Dig Endosc.

[15] Harada K, Kawano S, Hiraoka S, Kawahara Y, Kondo Y, Okada H (2015). Endoscopic radial incision and cutting method for refractory stricture of a rectal anastomosis after surgery. Endoscopy..

[16] Lee TH, Lee SH, Park JY, Lee CK, Chung IK, Kim HS (2009). Primary incisional therapy with a modified method for patients with benign anastomotic esophageal stricture. Gastrointestinal Endoscopy.

